# Modeling of Chilled/Supercooled Pork Storage Quality Based on the Entropy Weight Method

**DOI:** 10.3390/ani12111415

**Published:** 2022-05-30

**Authors:** Songsong Zhao, Hengxun Lin, Shuangqing Li, Chenghao Liu, Junhong Meng, Wenqiang Guan, Bin Liu

**Affiliations:** 1 Tianjin Key Laboratory of Refrigeration Technology, School of Mechanical Engineering, Tianjin University of Commerce, Tianjin 300134, China; 120200292@stu.tjcu.edu.cn (S.L.); lbtjcu@tjcu.edu.cn (B.L.); 2 Tianjin Key Laboratory of Food Biotechnology, School of Biotechnology and Food Science, Tianjin University of Commerce, Tianjin 300134, China; linhengxun@stu.tjcu.edu.cn (H.L.); liuchenghao1@stu.tjcu.edu.cn (C.L.); mengjunhong@stu.tjcu.edu.cn (J.M.)

**Keywords:** meat quality, shelf-life prediction, the entropy weight method, total viable counts

## Abstract

**Simple Summary:**

The quality of chilled meat is difficult to predict because many quality indexes need to be considered. The waste of meat resources caused by improper storage has caused huge economic losses in the meat industry. The entropy weight method (EWM) was widely used as an effective method of infusion of multiple attributes into a single index of food quality. In this study, the model based on the entropy weight method was used to predict and comprehensively evaluate the quality changes in chilled pork, and the relative error range between the measured and predicted shelf life was lower than 11%. The modeling based on EWM integrates the information from each quality index and provides accurate quality prediction, which will enable the food industry to enhance accurate judging of the shelf life and safety of meat.

**Abstract:**

The entropy weight method (EWM) was developed and used to integrate multiple quality indexes of pork to generate a comprehensive measure of quality. The Arrhenius equation and chemical kinetic reaction were used to fit and generate the shelf life prediction model. The pork was stored at the temperatures of 7 °C, 4 °C, 1 °C and −1 °C. Quality indexes, such as drip loss, color, shear force, pH, TAC, TVB-N and TBARS were measured. The results show that low temperatures effectively delay microbial growth and lipid oxidation. The regression coefficients (R^2^) for the comprehensive scores at each temperature were greater than 0.973 and the activation energy Ea was 9.7354 × 104 kJ mol^−1^. The predicted shelf life of pork stored at 7 °C, 4 °C, 1 °C and −1 °C was 4.35 d, 6.85 d, 10.88 d and 14.90 d, respectively. In conclusion, EWM is an effective method to predict the shelf life of chilled/supercooled pork.

## 1. Introduction

Pork is an important animal resource and plays a vital role in daily nutrition. China is the world’s largest pork producer and consumer, accounting for a quarter of the global total [1]. Whereas, the chemical interactions and microbial growth during transportation and storage constantly deteriorate pork, which reduces the quality of pork and results in economic loss [2].

Temperature is an important extrinsic factor of pork processing with impacts on pork quality and shelf life [3]. Chilling [4] (the storage temperature of 0 °C to 7 °C mostly), supercooling [5] (the storage temperature between the ice nucleation temperature and 0 °C) and freezing [6] (the storage temperature below −6 °C) are the most common storage methods. Normally, a lower storage temperature represents a longer shelf life of the meat, however, the freezing damage caused by too low a temperature would destroy the structure of muscle fibers and lead to high moisture loss and poor sensory quality after thawing [7], and result in resource waste and lower profitability. In order to maintain the original freshness of the meat, chilling and supercooling storage was most used. However, the effect of different chilling and supercooling temperatures on pork shelf life is limited by there being no suitable informational assessment method. The selection of suitable temperature is quite important in the control of meat quality and energy consumption during the storage and distribution processes [8].

The comprehensive quality of pork is determined by multiple quality parameters. The mass loss of pork during storage is primarily caused by the loss of water-holding capacity and reflected by drip loss [9], and the partial denaturation of myoglobin globulin occurs during storage, resulting in the color change of pork [10]. The deterioration of pork caused by microbial propagation and lipid oxidation increases the risk of edibility problems [11]. While the quality parameters are related to each other, the evaluation standard of each index is independent, which increases the difficulty of the comprehensive evaluation of pork quality. In previous research, the Arrhenius equation [12], WLF equation [13] and Michaelis–Menten model [14] were used to construct the shelf life prediction model, and the quality indicators, such as total volatile basic nitrogen (TVB-N), total aerobic count (TAC), and thiobarbituric acid-reactive substance (TBARS), were used to establish models to evaluate the quality changes [15]. However, a model depending on a single indicator cannot reflect the overall quality of the meat, however, predictive models based on a comprehensive evaluation of quality are limited in the literature.

Entropy was originally a thermodynamic concept and was later introduced into information theory to unify and reflect the changes in the information provided by each index [16]. The change in pork quality parameters caused by biochemical metabolic reactions and microbial propagation contain different amounts of information that can be reflected as entropy. The entropy weight method (EWM) is demonstrated to be a powerful tool for predicting the shelf life of various foods [17], which can avoid interference from human factors in the weighting of each evaluation index, and the evaluation results are more objective. Zou and Li [18]. applied the EWM to predict the shelf life of litchi at the temperature from 278 K to 293 K, however, it has not been reported where EWM was used as a comprehensive evaluation of meat quality during chilled/supercooled storage.

Thus, in this study, a novel model based on EWM is proposed to provide a comprehensive evaluation of pork quality during chilled/supercooled storage (7, 4, 1, and −1 °C), and the Arrhenius equation was fitted with reaction kinetics to construct an appropriate shelf-life prediction model and predict the quality change of pork, providing the theoretical and technical basis for pork storage. The changes between different storage states (chilled/supercooled) were compared and analyzed.

## 2. Materials and Methods

### 2.1. Meat Preparation and Treatment

Eight pigs (Duroc × Landrace × Yorkshire crossbred, 6 months of age, 85–95 kg live weight) with high health status were selected. After slaughter, 8 longissimus dorsi muscles were taken and transported to the laboratory in a portable incubator. After removing the surface fat and fascia, the muscles were divided into 64 rectangular pieces with a size of about 6 cm × 4 cm × 2 cm and a mass of about 40 g. The samples were randomly divided into four groups and stored in the freezer at 7, 4, 1 and −1 °C (supercooling temperature), respectively. The whole process was completed within 2 h. Samples were taken out every two days to test quality indices.

### 2.2. Analyses of Pork Quality Indices

#### 2.2.1. Determination of Drip Loss

A common method was used to measure drip losses of pork. The samples were allowed to moderate at 4 °C for 12 h prior to measurement and then the weights of the samples at this point were recorded as the final weights. Excess drip was wiped away with a paper towel. Drip loss of pork tenderloin samples was measured by comparing the final weights of samples (*M_T_*) stored under different conditions with the initial weights of the sample (*M*_0_), which is calculated using the Equation (1):

(1) $$Dirp\;loss(\%)=\frac{M_{0}-M_{T}}{M_{0}}\times 100$$

#### 2.2.2. Determination of Color

Four groups of samples were taken out from the polyethylene film and the color of the sample surface was measured using a handheld colorimeter (CR-400; Konica Minolta, Tokyo, Japan). Illuminant D65 was used. Before measurement, the instrument was calibrated with a White tile (C: Y = 93.5, x = 0.3114, Y = 0.3190). Each sample was tested four times, each time taking a different surface at random. The L*, a* and b* values were recorded and the results were averaged for data analysis.

#### 2.2.3. Determination of Shear Force

Shear force values of samples were measured according to the method of Qian et al. [8] with slight modifications. A texture Analyser (TA-XT Plus; Stable Micro System, Surrey, UK) with an HDP/BS blade was used to measure the shear force values. The pork sample (1 cm × 1 cm × 3 cm) was placed horizontally in the direction of the muscle fibers to facilitate vertical cutting with the blade. The pre-test speed and test speed were 1.50 mm/s with a distance of 25 mm, the post-test speed was 10.00 mm/s. The trigger force was 20 g. The maximum force to cut into the sample was recorded as the shear force. The test was repeated 3 times and the results were expressed in Newtons.

#### 2.2.4. Determination of Total Aerobic Count (TAC)

The total number of colonies in the samples was determined using the dilution plate method (Chinese National Standard GB4789.2-2016) [19]. In summary, 10 ± 0.1 g of beef sample was homogenized with nine volumes (*w*/*v*) of sterile water. The homogenate was pat with a flapping homogenizer for 2 min and serially diluted tenfold by adding 1 mL of homogenates to 9 mL of sterile water. The colony number was recorded after incubation at 37 °C for 48 h.

#### 2.2.5. Determination of Lipid Oxidation

Sample lipid oxidation measurements were made by thiobarbituric acid reactive substances (TBARS) using the method of Liu et al. [20] with minor adjustments. A sample of 10 g minced pork was homogenized in 20 mL of 20% trichloroacetic acid (TCA) using a homogenizer (Vortex2; IKA, Staufen, Germany) for 1 min, and then centrifuged at 5500 rpm for 15 min at 4 °C (H1850; Hunan Xiangyi centrifuge instrument Co., Ltd., Hunan, China). The supernatant was filtered through filter paper. Next, 5 mL of the filtrate was added to 5 mL of a 0.02 M solution of 2-thiobarbituric acid (TBA), and the blank treatment was 5 mL of 20% TCA mixed with 5 mL of TBA reagent. The solution was heated in a boiling water bath for 20 min. After cooling in tap water for 10 min, the absorbance of the solution at 532 nm was measured using a spectrophotometer (Evolution 201; Thermo Scientific, Waltham, MA, USA). The TBARS value was calculated by Equation (2):

(2) $$\mathrm{TBARS}(\mathrm{mg}/\mathrm{kg})=(A_{532}+0.002)\times 2.587$$

where *A*_532_ is the absorbance value of the assay solution.

#### 2.2.6. Determination of Total Volatile Basic Nitrogen (TVB-N)

TVB-N content of pork samples was evaluated by a semi-micro determination of nitrogen method according to Chinese National Standard GB 5009.228-2016 [21]. According to the standard, 20 ± 0.1 g of pork sample was weighed, 100 mL of distilled water was added, equilibrated for 30 min and then filtered. A total of 10 mL of filtrate and 5 mL of magnesium suspension (10 g/L) were added to the reaction chamber and distilled for 5 min. The distillate was collected in an Erlenmeyer flask containing 20 mL of boric acid aqueous solution (20 g/L) and three or four drops of an indicator being a mixture of 1 g/L methyl red-ethanol and 5 g/L bromocresol green. It was then titrated with 0.01 M hydrochloric acid standard titration solution. The value of TVB-N was calculated using Equation (3):

(3) $$\mathrm{TVB}\text{-}N(\mathrm{mg}/100g)=\frac{(v_{1}-v_{2})\times c\times 14}{m\times 10/100}\times 100$$

where *v*_1_ is the volume of HCl consumed by the pork sample (mL); *v*_2_ is the volume of HCl used to titrate the blank sample (mL); *c* is the concentration of HCl (mol/L); and *m* is the mass of the processed pork sample (g).

### 2.3. Modelling

#### 2.3.1. The Entropy Weight of Each Index

The changes in each quality index of pork samples during storage time of each treatment temperature are arranged in a matrix (*X_nm_*) and presented as follows:

(4) $$X_{nm}=\left[{}_{}\begin{array}{ccccccc} & G_{1} & G_{2} & \ldots & G_{j} & \ldots & G_{m}\\ U_{1} & x_{11} & x_{12} & \ldots & x_{1j} & \ldots & x_{1m}\\ U_{2} & x_{21} & x_{22} & \ldots & x_{2j} & \ldots & x_{2m}\\ \ldots & \ldots & \ldots & & \ldots & & \ldots \\ U_{i} & x_{i1} & x_{i2} & \ldots & x_{ij} & & x_{im}\\ \ldots & \ldots & \ldots & & \ldots & & \ldots \\ U_{n} & x_{n1} & x_{n2} & \ldots & x_{nj} & & x_{nm} \end{array}\right]$$

where *T*_1_, *T*_2_, *T*_3_, … *T_m_* is the m temperature treatments needed to be evaluated, *D*_1_, *D*_2_, *D*_3_, … *D_n_* is the value of *n* quality indices recorded in a certain time, *x_ij_* is the average of values on each tested day of *Di* for treatment *T_j_*, 0 < *i* ≤ *n*, 0 < *j* ≤ *m*.

The range of each index was different, thus, the value of each indicator was confined to the range of [0, 1] according to Equation (5) [16].

(5) $$r_{i}{}_{j}=\left\{ \begin{array}{l} \frac{\max\left\{X_{j}\right\}-X_{ij}}{\max\left\{X_{j}\right\}-\min\left\{X_{j}\right\}}\;\mathrm{benefit}\;\mathrm{type}\;\mathrm{index}\\ \frac{X_{ij}-\min\left\{X_{j}\right\}}{\max\left\{X_{j}\right\}-\min\left\{X_{j}\right\}}\;\cos t\;\mathrm{type}\;\mathrm{index} \end{array}\right.$$

Benefit type index means the higher the index value, the better the index; cost type index means the lower the index value, the better the index. After unification and normalization, the new evaluation matrix (*X*’*_nm_*) was established and shown in Equation (6).

(6) $$X_{nm}^{\prime }=\left[{}_{}\begin{array}{ccccccc} & G_{1} & G_{2} & \ldots & G_{j} & \ldots & G_{m}\\ U_{1} & r_{11} & r_{12} & \ldots & r_{1j} & \ldots & r_{1m}\\ U_{2} & r_{21} & r_{22} & \ldots & r_{2j} & \ldots & r_{2m}\\ \ldots & \ldots & \ldots & & \ldots & & \ldots \\ U_{i} & r_{i1} & r_{i2} & \ldots & r_{ij} & & r_{im}\\ \ldots & \ldots & \ldots & & \ldots & & \ldots \\ U_{n} & r_{n1} & r_{n2} & \ldots & r_{nj} & & r_{nm} \end{array}\right]$$

Then, the entropy of the quality index is calculated by Equations (7)–(9):

(7) $$H_{i}=-k\sum_{j=1}^{n}f_{ij}\ln f_{ij}$$

(8) $$f_{ij}=\frac{x_{ij}}{\sum_{j=1}^{n}x_{ij}}$$

(9) $$k=\frac{1}{\ln(n)}$$

where *f_ij_* = 0, define *f_ij_*ln(*f_ij_*) = 0.

The weight of each index is calculated according to the entropy, and the weight of each quality index is expressed as Equations (10) and (11):

(10) $$w_{i}=\frac{1-H_{i}}{\sum_{i=1}^{m}\left(1-H_{i}\right)}$$

(11) $$W_{i}={\left(w_{i}\right)}_{1\times n}^{T}$$

#### 2.3.2. The Calculation of Comprehensive Quality

The comprehensive quality of different stored pork on each test day was calculated by Equation (12):

(12) $$Q=W_{i}\times {X^{\prime \prime }}_{nm}$$

#### 2.3.3. Chemical Reaction Kinetics

By selecting the characteristic indices of pork quality degradation and combining them with the chemical reaction kinetics theory, the quality decay function can be fitted, and then the kinetic model of chemical quality change can be constructed, so as to predict the food shelf life. Zero-order and first-order reaction models are widely used [18].

Zero-order reaction models:

(13) $$C=kt+C_{0}$$

First-order reaction models:

(14) $$C=C_{0}e^{kt}$$

where *t* is the storage time, *C*_0_ is the initial quality value, *C* is the quality value on the day *t*, and *k* is the quality decay rate.

#### 2.3.4. Arrhenius Equation

According to the fitting effect of the logarithmic points of the kinetic model, the order is determined and the reaction rate constant *K* is obtained. The relationship between kinetic constant *K* and storage temperature *T* can be expressed by Arrhenius equation:

(15) $$\ln k=\ln k_{0}-\frac{E_{a}}{RT}$$

where *k*_0_ is the frequency factor, *R* is the universal gas constant, and *E_a_* is the activation energy, which is calculated by regressing the value of ln*k* and the reciprocal of the storage temperature.

By linear fitting ln*k* and 1/*T*, *Ea* and *A* values can be obtained according to slope and intercept. By substituting Equation (15) into Equations (16) and (17), the quality prediction model of pork in zero-grade and first-grade reactions can be obtained:

(16) $$SL_{0}=\frac{C-C_{0}}{k_{0}e^{-\frac{E_{0}}{RT}}}$$

(17) $$SL_{1}=\frac{\ln\frac{C}{C_{0}}}{k_{0}e^{-\frac{E_{0}}{RT}}}$$

where *SL*_0_ is the quality prediction model of pork under zero-order reaction. *SL*_1_ is the quality prediction model of pork under first-order reaction.

The relative errors between the measured and predicted values served as the criteria for evaluating the prediction accuracy of the models.

### 2.4. Statistical Analysis

Excel 2010 (v.2010, Microsoft Inc., Redmond, WA, USA) and Origin (v.9.1, OriginLab Corp., Hampton, VA, USA) were used for data sorting and image processing. The analysis of variance (ANOVA) using SPSS software (v.26.0, IBM, Chicago, IL, USA), and significance was defined at *p* < 0.05 while Duncan’s multiple range test was used to determine differences between treatment means.

## 3. Results

### 3.1. Analyses of Pork Quality Indices

#### 3.1.1. Drip Loss

**Figure 1:**
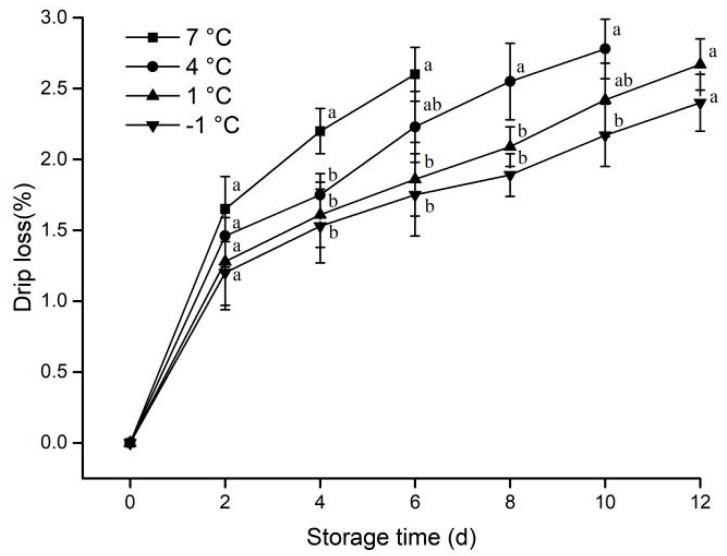
Effect of different storage temperatures on drip loss of pork. Different lowercase letters (a, b) indicate significant difference (*p* < 0.05) between each treatment on the same storage time.

Moisture is the most important component of muscle and accounts for about 75% of muscle composition [6]. The drip loss is highly related to the edible mass and affects the economic return of the meat industries [22]. The drip loss of the stored pork at the different temperatures is presented in Figure 1.

The drip loss of four groups of samples subjected to different treatments increased with the extension of the storage period. The drip loss was lower as the storage temperature decreased. After the fourth day, the drip loss of the samples stored at 1 °C and −1 °C was significantly lower compared with that of the samples stored chilled at 7 °C (*p* < 0.05). The result was consistent with that of Lin et al. [23].

#### 3.1.2. Color

The color changes of pork are the result of microbial contamination and the degree of myoglobin oxidation and will determine consumer acceptability of the stored meat [24].

**Figure 2:**
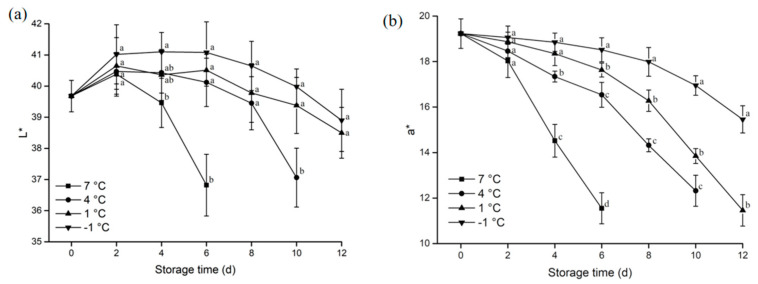
(**a**) Effect of different storage temperatures on L* value of pork; (**b**) Effect of different stored temperatures on a* value of pork. Different lowercase letters (a−d) indicate significant difference (*p* < 0.05) between each treatment on the same storage time.

As shown in Figure 2a, the brightness (L*) of the four groups of samples under different treatments increased by the second day and then decreased as the storage period increased. The L* value of the samples at −1 °C and 1 °C were significantly higher than that of 7 °C samples (*p* < 0.05) after the sixth day of storage. The lower storage temperatures inhibited the oxidation of myoglobin in pork samples, which led to a more gradual reduction in the brightness (L*) of the pork samples.

However, the redness (a*) of the samples decreased with the extension of the storage period. The lower the storage temperature, the higher the redness (a*) value. Throughout the storage process, as the degree of oxidation increased [25], the color of the samples gradually turned dark brown, and the a* value decreased. The redness of samples stored at −1 °C and 1 °C was significantly higher than that at 7 °C from the fourth day of storage (*p* < 0.05). However, from the eighth day onwards, the redness of the samples stored at −1 °C was significantly different from 1 °C (*p* < 0.05).

#### 3.1.3. Shear Force

**Figure 3:**
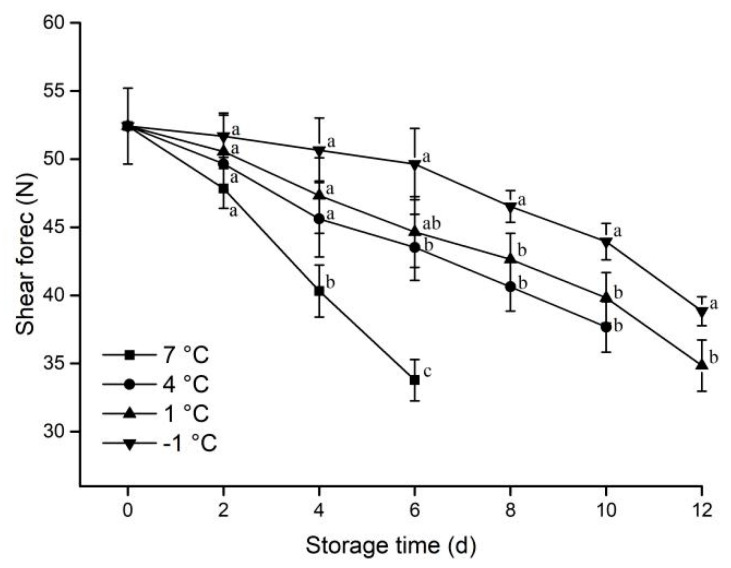
Effect of different storage temperatures on shear force of pork. Different lowercase letters (a−c) indicate significant difference (*p* < 0.05) between each treatment on the same storage time.

Tenderness determines the palatability of meat, and this in turn plays an important role in human sensory perception of pork quality [26]. The shear force changes during the storage of each treatment pork is shown in Figure 3.

Shear values decreased with increasing storage time for the four treatment groups. The rate of decline in shear values was significantly higher for samples stored at 7 °C than for the other groups. The lower the storage temperature, the slower the shear force decrease. From the fourth day of storage, the shear values of the samples at −1 °C and 1 °C were significantly higher than that at 7 °C. From the eighth day of storage, the shear values of the samples at −1 °C were also significantly higher than that at 1 ℃. It was shown that the lower temperature inhibited the deformation of the proteins in the pork samples to slow down the reduction in shear values [8]. The result is consistent with that of You et al. [27].

#### 3.1.4. TAC

Meat is rich in trace elements and high-value protein and other bioactive compounds, which provides a very suitable medium for microbial growth [11]. The growth of microbial in meat may lead to an increase in foodborne diseases [28]. The Chinese National Food Safety Standard (GB 2707-2016, 2016) [19] stipulates that microbes in meat cannot exceed 6 log (CFU/g).

**Figure 4:**
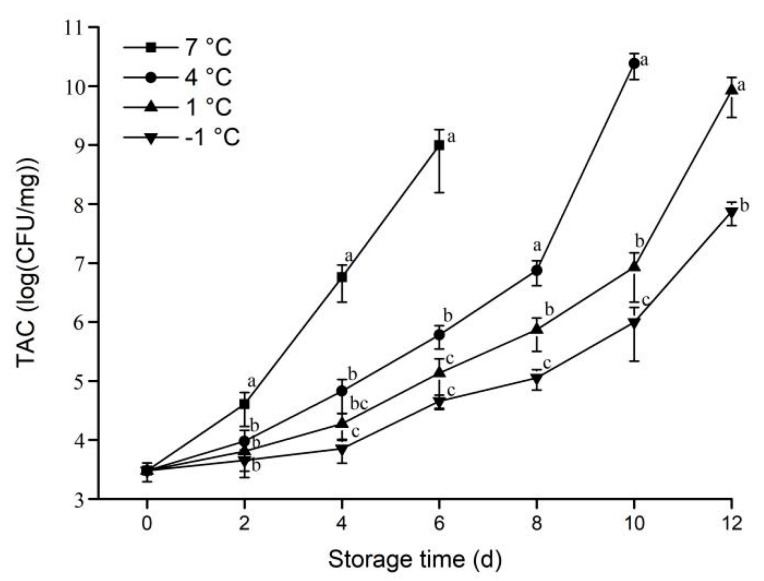
Effect of different storage temperatures on TAC of pork. Different lowercase letters (a−c) indicate significant difference (*p* < 0.05) between each treatment on the same storage time.

As shown in Figure 4, at the higher storage temperature (7 °C), the TCA of the samples increased at a faster rate during the storage period compared to the other groups. From the second day onwards, the TAC at −1 °C and 1 °C were significantly lower than samples under storage at 7 °C and with the extension of the storage period, the differences further increased. The TAC did not increase significantly in the samples stored at −1 °C, 1 °C and 4 °C during 0 to 4 days of storage, the microbial growth rate of pork was lower at a lower storage temperature.

#### 3.1.5. Lipid Oxidation

During storage, the barbiturate acid-reactive substances are produced by lipid oxidation in meat. The barbiturate acid-reactive substances can be used to reflect the degree of meat lipid oxidation [27].

**Figure 5:**
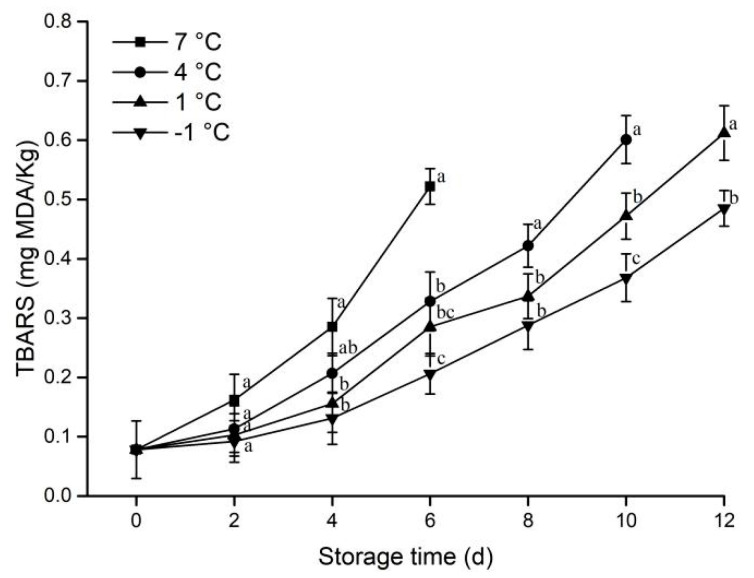
Effect of different storage temperatures on TBARS of pork. Different lowercase letters (a−c) indicate significant difference (*p* < 0.05) between each treatment on the same storage time.

As shown in Figure 5, the TBARS value increased continuously in all groups during storage. The TBARS value at 7 °C after the sixth day was significantly higher than that of other samples (*p* < 0.05), and the lower the temperature, the smaller the TBARS value. This result indicated that low temperature inhibits lipid oxidation. From the tenth day of storage, the TBARS value at −1°C was significantly lower than that at 1 °C (*p* < 0.05).

#### 3.1.6. TVB-N

**Figure 6:**
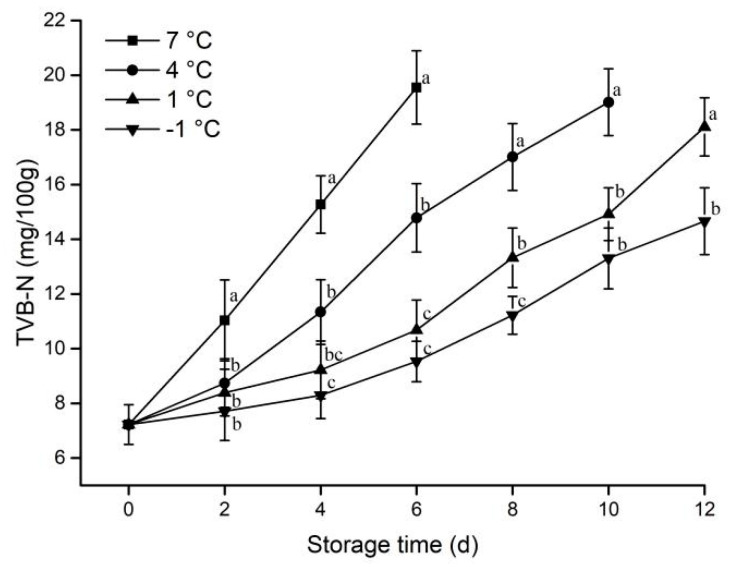
Effect of different storage temperatures on TVB-N of pork. Different lowercase letters (a−c) indicate significant difference (*p* < 0.05) between each treatment on the same storage time.

The accumulation of ammonia and amines by protein denaturation and microbial growth results in higher total volatile basic nitrogen (TVB-N) concentration. The TVB-N value can be used to reflect the freshness of meat [28]. According to The Chinese National Food Safety Standard (GB 2707-2016, 2016) [21], if the TVB-N values of the meat exceed 15 mg/100 g, it is considered to be a deteriorated product. The change in TVB-N values during storage for each treatment pork is shown in Figure 6.

The TVB-N concentration of each treatment group increased with storage time. The TVB-N values of samples stored at 7 °C from the second day of storage had significantly higher than that at −1 °C and 1 °C. This indicated that the TVB-N values of the pork samples were heavily influenced by temperature. After 12 days of storage at −1 °C, the TVB-N values remained below 15 mg/100 g, which prolongs the storage life of the samples by 8 days compared with the 7 °C samples.

### 3.2. Performance of Model

#### 3.2.1. Evaluation Matrix of Chilled/Supercooled Pork

The changes in drip loss of different stored pork for each day were arranged in matrix (*X_nm_*), and the evaluation matrix (*X_nm_*) after unification and normalization calculation is given in matrix (19), The calculation process of other indices is similarly determined using Equation (5).

(18) $$X_{nm}=\left[\begin{array}{ccccc} \mathrm{Storage}\;\mathrm{time}\;(d) & 7\;{}^{\circ}C & 4\;{}^{\circ}C & 1\;{}^{\circ}C & -1\;{}^{\circ}C\\ 0 & 0.000 & 0.000 & 0.000 & 0.000\\ 2 & 1.650 & 1.460 & 1.280 & 1.200\\ 4 & 2.200 & 1.750 & 1.610 & 1.530\\ 6 & 2.600 & 2.230 & 1.860 & 1.750\\ 8 & & 2.550 & 2.090 & 1.890\\ 10 & & 2.780 & 2.420 & 2.170\\ 12 & & & 2.670 & 2.400 \end{array}\right]$$

(19) $${X^{\prime }}_{nm}=\left[\begin{array}{ccccc} \mathrm{Storage}\;\mathrm{time}\;(d) & 7\;{}^{\circ}C & 4\;{}^{\circ}C & 1\;{}^{\circ}C & -1\;{}^{\circ}C\\ 0 & 1.000 & 1.000 & 1.000 & 1.000\\ 2 & 0.406 & 0.475 & 0.540 & 0.568\\ 4 & 0.209 & 0.371 & 0.421 & 0.450\\ 6 & 0.065 & 0.198 & 0.331 & 0.371\\ 8 & & 0.083 & 0.248 & 0.320\\ 10 & & 0.000 & 0.129 & 0.219\\ 12 & & & 0.040 & 0.137 \end{array}\right]$$

The new evaluation matrix (*X”_nm_*) included all changes of the indices and is shown in matrix (20)

(20) $${X^{\prime \prime }}_{nm}=\left[\begin{array}{ccccccccc} \mathrm{Temperature}\;({}^{\circ}C) & \mathrm{Time}\;(d) & \mathrm{Drip}\;\mathrm{loss} & L* & a* & \mathrm{Shear}\;\mathrm{force} & \mathrm{TAC} & \mathrm{TVB}\text{-}N & \mathrm{TBARS}\\ & & 1.000 & 1.000 & 1.000 & 1.000 & 1.000 & 1.000 & 1.000\\ & 0 & 1.000 & 0.668 & 1.000 & 1.000 & 1.000 & 1.000 & 1.000\\ 7 & 2 & 0.406 & 0.832 & 0.846 & 0.755 & 0.888 & 0.691 & 0.843\\ & 4 & 0.209 & 0.617 & 0.394 & 0.351 & 0.529 & 0.347 & 0.612\\ & 6 & 0.065 & 0.000 & 0.012 & 0.000 & 0.180 & 0.000 & 0.169\\ & & 1.000 & 1.000 & 1.000 & 1.000 & 1.000 & 1.000 & 1.000\\ & 0 & 1.000 & 0.668 & 1.000 & 1.000 & 1.000 & 1.000 & 1.000\\ & 2 & 0.475 & 0.853 & 0.901 & 0.851 & 0.906 & 0.877 & 0.934\\ 4 & 4 & 0.371 & 0.843 & 0.757 & 0.635 & 0.818 & 0.666 & 0.758\\ & 6 & 0.198 & 0.771 & 0.654 & 0.523 & 0.668 & 0.387 & 0.532\\ & 8 & 0.083 & 0.614 & 0.368 & 0.367 & 0.503 & 0.206 & 0.356\\ & 10 & 0.000 & 0.056 & 0.111 & 0.209 & 0.000 & 0.044 & 0.021\\ & & 1.000 & 1.000 & 1.000 & 1.000 & 1.000 & 1.000 & 1.000\\ & 0 & 1.000 & 0.688 & 1.000 & 1.000 & 1.000 & 1.000 & 1.000\\ & 2 & 0.540 & 0.895 & 0.954 & 0.900 & 0.951 & 0.905 & 0.953\\ & 4 & 0.421 & 0.827 & 0.887 & 0.726 & 0.872 & 0.838 & 0.854\\ 1 & 6 & 0.331 & 0.862 & 0.794 & 0.583 & 0.735 & 0.719 & 0.612\\ & 8 & 0.248 & 0.692 & 0.620 & 0.476 & 0.648 & 0.505 & 0.515\\ & 10 & 0.129 & 0.598 & 0.308 & 0.324 & 0.488 & 0.376 & 0.262\\ & 12 & 0.040 & 0.393 & 0.000 & 0.057 & 0.114 & 0.117 & 0.000\\ & & 1.000 & 1.000 & 1.000 & 1.000 & 1.000 & 1.000 & 1.000\\ & 0 & 1.000 & 0.688 & 1.000 & 1.000 & 1.000 & 1.000 & 1.000\\ & 2 & 0.568 & 0.981 & 0.978 & 0.960 & 0.978 & 0.960 & 0.974\\ & 4 & 0.450 & 1.000 & 0.951 & 0.905 & 0.941 & 0.913 & 0.901\\ 1 & 6 & 0.371 & 0.995 & 0.909 & 0.850 & 0.834 & 0.813 & 0.760\\ & 8 & 0.320 & 0.895 & 0.840 & 0.683 & 0.739 & 0.676 & 0.607\\ & 10 & 0.219 & 0.738 & 0.707 & 0.546 & 0.612 & 0.507 & 0.457\\ & 12 & 0.137 & 0.486 & 0.515 & 0.271 & 0.431 & 0.397 & 0.238 \end{array}\right]$$

#### 3.2.2. The Entropy Weight of Each Index and the Comprehensive Quality of Pork

The entropy weights of drip loss, L*, a*, shear force, TAC, TVB-N and TBARS are calculated and used to compose the entropy weight matrix *W_i_* (21) using Equations (7)–(11).

(21) $$W_{i}=\left[\begin{array}{ccccccc} \mathrm{Drip}\;\mathrm{loss} & L* & a* & \mathrm{Shear}\;\mathrm{force} & \mathrm{TAC} & \mathrm{TVB}\text{-}N & \mathrm{TRARS}\\ 0.1500 & 0.1046 & 0.1475 & 0.1533 & 0.1304 & 0.1604 & 0.1539 \end{array}\right]$$

The comprehensive quality changes of pork during storage time are shown in Table 1. The comprehensive quality of pork in each treatment decreased with the extension of storage time, and the rate decreased with the increase of storage temperature, which is consistent with the changes in pork quality after slaughter.

According to the entropy weight matrix *W_i_*, the TVB-N values play the most important part in reflecting the freshness of pork. Therefore, with a TVB-N of 15 mg/100 g being the accepted marker of the lower limit for acceptable pork quality [21], the equivalent comprehensive evaluation quality marker value was 0.38

#### 3.2.3. Kinetic Analysis

Most of the decay functions of the pork quality index correspond with a zero- or one-order kinetic reaction model [18]. The zero- and first-order kinetic reaction rate constants k and coefficient of determination R^2^ of pork comprehensive quality were determined with the measured comprehensive evaluation quality value and calculated by Equations (14) and (15) and presented in Table 2. According to the result of R^2^, the zero-order kinetic quality index was used to establish the shelf life predicted model.

#### 3.2.4. Establish of Model

According to the comprehensive quality change rate of pork at 7, 4, 1 and −1 °C, a linear fitting was carried out using Equation (16), with *1/T* as the abscissa, ln (*−k*) is used as the vertical standard for comprehensive evaluation quality, therefore, model parameters such as activation energy *E_a_* and pre-exponential factor *K*_0_ were obtained and are shown in Table 3.

Putting the results from Table 3 into Equation (16) to obtain the pork shelf-life model, the results are as follows:

(22) $$SL_{CQ}=\frac{C_{0}-C_{i}}{-1.877\times 10^{17}e^{-\frac{9.7354\times 10^{4}}{8.314T}}}$$

(23) $$SL_{TVB\text{-}N}=\frac{C_{0}-C_{i}}{1.4247\times 10^{22}e^{-\frac{1.17179\times 10^{5}}{8.314T}}}$$

(24) $$SL_{TBARS}=\frac{C_{0}-C_{i}}{7.74768\times 10^{8}e^{-\frac{7.74768\times 10^{8}}{8.314T}}}$$

(25) $$SL_{TAC}=\frac{C_{0}-C_{i}}{9.57795\times 10^{17}e^{-\frac{9.7175\times 10^{4}}{8.314T}}}$$

where the *SL_CQ_* is the shelf-life predicted model based on the comprehensive quality of pork, the *SL_TVB-N_* is the shelf-life predicted model based on the comprehensive quality of pork, the *SL_TBARS_* is the shelf-life predicted model based on the comprehensive quality of pork, the *SL_TAC_* is the shelf-life predicted model based on the comprehensive quality of pork, *C*_0_ is the final value of the index in pork at the end of the shelf life, *C_i_* is the initial value of the index in pork, *T* is the storage temperature.

#### 3.2.5. Validation of Model

The relative error between the predicted shelf life and the measured shelf life is shown in Table 4. The relative error of the comprehensive quality predicted model was 5.09%, which is lower than that of other models based on a single index. These results indicated that the comprehensive evaluation quality model could predict the shelf life of pork under conditions of 7~−1 °C.

## 4. Discussion

Among the many factors that affect fresh meat, temperature is one of the most important [23]. In general, lower temperatures support a longer shelf life of meat, our results also confirm this viewpoint (Table 4), the shelf life of pork was prolonged with the decrease in storage temperature. However, ice formation occurs at very low storage temperatures leading to freezing damage, resulting in decreased meat quality [29]. Thus, it is important to choose a suitable temperature. Supercooling storage can preserve meat under subzero conditions avoiding the freezing damage caused by ice crystallization and inhibiting the quality deterioration [30]. Supercooling preserved food at temperatures 1–2 °C below their initial freezing point temperature (−0.5 °C to −2.8 °C for most food) and without the formation of ice crystals [31]. Stonehouse et al. [32] reported that the ice point of pork was about −1.5 °C. Our result also confirmed this finding, −1 °C is a suitable storage temperature, which maintained the stable supercooled state of pork. The effect of a supercooled temperature (−1 °C) significantly delays the quality change rate of pork compared with a chilled temperature (7, 4, and 1 °C).

Each of the meat quality indexes, such as physicochemical index, sensory index, and microbiological index, are related to each other [33]. Leygonie et al. [34] found that the heme in myohemoglobin may be released into the extracellular space following the increase in temperature, resulting in color changes. The TVB-N of meat is highly related to microbial growth, with the ammonia and amine-derived products of microbial growth leading to an increased TVB-N [35]. Thus, a single index makes it difficult to reflect the quality of meat. The entropy weight method (EWM), which is based on the statistical physics and the concept of entropy in thermodynamics, can effectively avoid the man-made interference in the weighting of each evaluation indices and makes the calculation results more objective [16]. The multiple evaluation index can be merged into a comprehensive evaluation index using the EWM. In this research, the entropy weight of each index calculated by the EWM was similar to those of previous studies, and the comprehensive evaluation index is suitable for the kinetics and Arrhenius model because it is time-structured.

The Arrhenius equation, combined with the kinetic model, is widely used to predict the shelf life of food [36], however, the temperatures over which the equation applies are of limited range [37]. Most of the models consider only one or two quality indicators and fail to account for the true internal reactions, however, it is difficult to control the accuracy of a model with more parameters. In this paper, changes in the comprehensive evaluation indices based on the EWM fitted well to the zero-order kinetics model at different temperatures, the average relative error of the model was only 5.09%, which means that the Arrhenius equation combined with the kinetic model based on EWM was able to predict the shelf life of pork at the temperature range from 7 °C to −1 °C.

## 5. Conclusions

This experiment explored the effects of different shelf temperatures (7, 4, 1 and −1 °C,) on the storage quality of pork. The results showed that low-temperature conditions of 1 and −1 °C delayed the rate of drip loss and limited the rate of TVB-N production along with both inhibiting microbial growth and lipid oxidation. Compared with 7 °C and 4 °C, −1 °C reduced the rate of quality deterioration and effectively extends the shelf life of pork to more than 12 d. In addition, the evaluated method of comprehensive quality pork based on EWM was established, and the Arrhenius equation was combined with a chemical kinetic reaction to predict the shelf life of pork. The correlation coefficient of the comprehensive quality predicted model was 0.9734, and the average relative error was 5.09%. The comprehensive pork quality prediction model is an effective method to predict the shelf life of chilled/supercooled pork.

## Figures and Tables

**Table 1:** Comprehensive quality change of pork at each temperature during storage.

Storage Time (d)	7 °C	4 °C	1 °C	−1 °C
0	0.9652	0.9652	0.9653	0.9653
2	0.7445	0.8263	0.8690	0.9107
4	0.4265	0.6829	0.7712	0.8588
6	0.0608	0.5178	0.6517	0.7801
8		0.3406	0.5188	0.6684
10		0.0644	0.3413	0.5294
12			0.0894	0.3452

**Table 2:** Zero- and first-order kinetic reaction rate constants k and coefficient of determination R^2^.

Quality Index	Reaction Order	Storage Temperature (°C)	Reaction Rate *k*	Determination R^2^	The Average Values of R^2^
TVB-N	Zero-order	7 °C	1.99083 ± 0.0631	0.9411	0.8391
		4 °C	1.09055 ± 0.1828	0.6366	
		1 °C	0.68361 ± 0.14064	0.8189	
		−1 °C	0.43758 ± 0.14999	0.9597	
	First-order	7 °C	0.18838 ± 0.01874	0.9982	0.4452
		4 °C	0.10872 ± 0.00877	0.2342	
		1 °C	0.0695 ± 0.0065	0.0305	
		−1 °C	0.04421 ± 0.01008	0.5178	
TBARS	Zero-order	7 °C	0.05591 ± 0.01339	0.9516	0.9428
		4 °C	0.03734 ± 0.0178	0.9302	
		1 °C	0.03046 ± 0.01110	0.9142	
		−1 °C	0.02179 ± 0.009227	0.9753	
	First-order	7 °C	0.33541 ± 0.02144	0.8571	0.3472
		4 °C	0.21994 ± 0.02363	0.2322	
		1 °C	0.17779 ± 0.02742	0.0378	
		−1 °C	0.13395 ± 0.02895	0.2618	
TAC	Zero-order	7 °C	0.71860 ± 0.2384	0.914	0.8902
		4 °C	0.42991 ± 0.1386	0.7602	
		1 °C	0.31190 ± 0.1082	0.9033	
		−1 °C	0.19892 ± 0.0888	0.9834	
	First-order	7 °C	0.14841 ± 0.0300	0.6874	0.5329
		4 °C	0.08713 ± 0.0037	0.4289	
		1 °C	0.0629 ± 0.0095	0.3325	
		−1 °C	0.0435 ± 0.01486	0.6827	
Comprehensive scores	Zero-order	7 °C	−0.13193 ± 0.0166	0.9861	0.9176
		4 °C	−0.07656 ± 0.0074	0.8631	
		1 °C	−0.05669 ± 0.0087	0.8942	
		−1 °C	−0.03619 ± 0.0091	0.9268	
	First-order	7 °C	−0.26490 ± 0.1418	0.9200	0.6118
		4 °C	−0.09956 ± 0.0200	0.3456	
		1 °C	−0.06294 ± 0.0097	0.5032	
		−1 °C	−0.04273 ± 0.0201	0.6784	

**Table 3:** The parameter of prediction model for the shelf-life.

Quality Index	The Initial Value	The Final Value	Kinetic Order	Pre-Exponential Factor *K*_0_	Activation Energy *E_a_*	*R* ^2^
TVB-N	7.22	15.0	Zero-order	1.42470 × 10^22^	1.1718 × 10^5^	0.9944
TBARS	0.078	0.5	Zero-order	7.74768 × 10^8^	7.0484 × 10^4^	0.9221
TAC	3.3	6.0	Zero-order	9.57795 × 10^17^	9.7175 × 10^4^	0.9779
Comprehensive scores	0.9652	0.4	Zero-order	1.87700 × 10^17^	9.7354 × 10^4^	0.9734

**Table 4:** Experimental and predicted quality values under different temperatures.

Quality Index	Storage Temperature (°C)	Predict Shelf Life (d)	Measured Shelf Life (d)	Relative Error (%)	Average Relative Error (%)
TVB-N	7 °C	3.95	3.93	5.09	8.65
	4 °C	6.85	6.33	7.35	
	1 °C	11.87	10.93	8.53	
	−1 °C	17.32	14.76	17.41	
TBARS	7 °C	7.66	3.93	94.90	56.28
	4 °C	10.63	6.33	67.84	
	1 °C	14.86	10.93	35.91	
	−1 °C	18.66	14.76	26.45	
TAC	7 °C	3.78	3.93	3.89	9.17
	4 °C	5.95	6.33	6.29	
	1 °C	9.42	10.93	13.86	
	−1 °C	12.89	14.76	12.64	
Comprehensive quality	7 °C	4.35	3.93	10.79	5.09
	4 °C	6.85	6.33	8.11	
	1 °C	10.88	10.93	0.53	
	−1 °C	14.90	14.76	0.93	

## Data Availability

The data presented in this study are available on request from the corresponding author.
